# Health-related quality of life and associated factors among people living with HIV/AIDS in Sichuan, China: A cross-sectional study

**DOI:** 10.3389/fpubh.2023.1133657

**Published:** 2023-03-13

**Authors:** Hua Zhong, Fuling Wei, Yuqing Song, Hong Chen, Zhao Ni

**Affiliations:** ^1^West China School of Nursing, West China Hospital, Sichuan University, Chengdu, Sichuan, China; ^2^Center for Interdisciplinary Research on AIDS (CIRA), Yale University, New Haven, CT, United States; ^3^School of Nursing, Yale University, New Haven, CT, United States

**Keywords:** HIV, health-related quality of life, China, influencing factors, cross-sectional study

## Abstract

**Purpose:**

This study aimed to explore health-related quality of life (HRQoL) and its associated factors among people living with HIV/AIDS (PLWH) in Sichuan, China.

**Methods:**

A total of 401 PLWH were recruited from the city of Panzhihua between August 2018 and January 2019. Demographic characteristics and disease-related data were collected by self-administered questionnaires and medical system records. Health-related quality of life (HRQoL) was measured by the medical outcome study HIV health survey (MOS-HIV), which measured ten subdimensions and two summarized dimensions, the physical health summary score (PHS) and the mental health summary score (MHS). Logistic regression models were used to explore the variables independently associated with quality of life.

**Results:**

The PHS and MHS measured by MOS-HIV were 53.66 ± 6.80 and 51.31 ± 7.66, respectively. Younger age, higher educational level, no methadone use, higher CD4 lymphocyte counts, less symptom counts and heathy BMI significantly were associated with higher HRQOL in the univariate χ^2^-test analysis. Education level was found to have a significant influence on patients' quality of life, both in physical health (*P* = 0.022) and mental health (*P* = 0.002) dimensions. Younger age (*P* = 0.032), higher CD4 lymphocyte counts (*P* = 0.007), less symptom counts (*P* < 0.001) and health BMI level (*P* < 0.001) were positively related to the PHS of quality of life in the multivariable logistic regression model.

**Conclusion:**

The HRQoL of PLWH in Sinchuan Province was relatively low. Age, educational level, methadone use, CD4 lymphocyte counts, symptom counts and BMI were positively related to quality of life. This study indicates that health caregivers should pay more attention to comorbidity issues and mental health in PLWH, especially for those with lower education levels, unhealthy body mass index, more symptomatic presentation and older age.

## Introduction

The human immunodeficiency virus/acquired immunodeficiency syndrome (HIV/AIDS) pandemic is a serious global challenge (1). According to the Joint United Nations Program on HIV/AIDS (UNAIDS), there were 37.7 (30.2–45.1) million people living with HIV/AIDS (PLWH), and 36.3 (27.2–47.8) million people died from AIDS-related illnesses worldwide by the end of 2021 (1). In China, there are 1.147 million people living with HIV/AIDS (2).

The government of China has implemented the “Four Free and One Care” policy since 2003 in response to the HIV/AIDS epidemic (3). It is well known that one of the “Four Free” was to provide free highly active antiretroviral therapy (HAART) for all PLWH who meet the criteria for antiviral treatment. They can obtain free antiviral medication and health counseling every 3 months at the outpatient department of the infectious disease hospital or a general hospital with an infectious disease department. Thanks to the development of clinical treatment techniques and HAART, AIDS has transformed from a fatal infectious disease to a manageable chronic illness (4). The use of HAART in PLWH has substantially decreased the morbidity and lethality caused by HIV/AIDS symptoms (5), boosted their immune system (6), and improved their health-related quality of life (HRQoL) (7). A new “beyond viral suppression” model suggested adding a “fourth 95” to the UNAIDS' 95–95–95 target to ensure that 95% of PLWH with suppression have good HRQoL (8–10).

HRQoL is a multidimensional concept that includes dimensions such as physical health, psychological health, social functioning, and perception of general health (11). It emphasizes the importance of an overall subjective feeling of wellbeing pertaining to aspects of morale, happiness and satisfaction. Changes in HRQoL, including functional status and personal perceptions, may last throughout the rest of the PLWH's life (12).

Many previous studies around the world have proven that social problems such as cultural beliefs (13), sociodemographic characteristics (14, 15), socioeconomic characteristics (15–17), presence of comorbidities (18), stage of the disease (19), psychological (20), and clinical factors (18) can affect HRQoL among PLWH. Furthermore, alcohol drinking (18), depression (21), and spiritual belief in their disease and medication can also affect the mental and physical aspects of HRQoL (20, 22). Although the HRQoL of PLWH has significantly improved after treatment with HAART (9), drug-related side effects (15), poor adherence to HAART (18), and irregular medical follow-up have impaired HRQoL (15).

Many studies (20, 23–25) have explored the determinants of HRQoL in PLWH in China and found that older age, single, unemployment, low education, living in rural areas, and low CD4+ cell count are associated with low HRQoL among PLWH in China. Sichuan Province has one of the highest incidences of HIV/AIDS in China (26). Since the first AIDS case was reported in Sichuan in 1991, the province has reported more than 110,000 surviving HIV infections/patients as of 2017 (ranking first in China), with a 0.13% survival rate (ranking fourth in China). The number of new cases in the province continues to grow, ranking first in China for five consecutive years (27). However, there was a scarcity of studies reporting the HRQoL of PLWH. In the literature, several instruments, including the Medical Outcomes Study 36-item short-form health survey (28), 12-item short-form health survey (29), Medical Outcomes Study HIV Health Survey (MOS-HIV) and World Health Organization quality of life (WHOQOL)-BREF (30), have been used to evaluate the HRQoL of PLWH, but MOS-HIV is the most widely used measurement to assess HRQoL for PLWH with well-established psychometric properties (31).

Knowing the determinants of HRQoL would enable PLWH, their families, healthcare providers and policy makers to develop relevant and holistic interventions to improve the general wellbeing and overall HRQoL of PLWH (32). In particular, HRQoL in PLWH is critical for monitoring the impact of drug therapy on disease progression (33). Thus, the aim of this study was to investigate HRQoL status and associated factors in PLWH by using the MOS-HIV.

## Methods

### Study design and participants

This was a cross-sectional study conducted from August 2018 to January 2019 in Panzhihua city, Sichuan Province, China. HRQoL was used as the main indicator, and the sample content was designed to be 328 with reference to the relevant literature and the mean survey formula. The required sample size was finally determined to be 410 cases because 20% of invalid questionnaires were considered. Regarding the sites for conducting the sampling, 2 districts or counties in Panzhihua were randomly selected to conduct the survey. Finally, Renhe District and Miyi County were chosen. The researcher inquired about the number of PLWH in different districts or counties of Panzhihua City in that year to calculate the relative proportion to determine the sample size assigned to the two sites. And the convenient sampling sites were selected as the Fourth People's Hospital of Renhe District and the People's Hospital of Miyi County in Panzhihua City, respectively, both of which were designated as PLWH getting free medications, testing and consulting. Participants were recruited through convenience sampling. The inclusion criteria of participants were (1) HIV seropositivity, (2) age older than 17 years, (3) willing to fill out the study questionnaire. People with mental illness or cognitive impairment were excluded.

### Ethical considerations

The study was approved by the West China Hospital Medical Ethics Committee (study ID#: 20170430). The objectives and procedures of the study were verbally explained to participants. Before the study, participants signed written informed consent.

### Data collection

All data were collected through paper questionnaires. First, the investigators received training about the administration of the questionnaires. Second, all participants were informed of the purpose, content and potential risks of the study before the investigation. Participants independently completed the anonymous questionnaires. If the participants had difficulty understanding or reading the questionnaire, the investigators explained it in detail and recorded the answer if inquired. After the questionnaire was completed by participants recruited by the local CDC, the investigators immediately completed the questionnaire in person to verify whether the respondent had answered all the questions. If the patient refused to answer and the incomplete part of the questionnaire was >20%, the questionnaire was discarded. Each participant was paid ¥50 (equivalent to $7) as compensation for transportation.

### Measurements

#### Sociodemographic characteristics

Sociodemographic characteristics included sex, age, ethnicity, educational level, marital status, religion, sexual orientation, religious beliefs, occupation, alcohol consumption, residence, smoking habits, drinking habits, BMI and per capita monthly household income. In this study, age was divided into three groups: young adults aged 18–45 years, middle-aged adults aged 45–60 years and elderly adults aged 65 years and above. Participants were classified into 4 categories according to the weight determination criteria for Chinese adults published by the National Health and Wellness Commission Participations, which were underweight (<18.5), healthy weight (18.5–24.9), overweight (25–27.9), and obese (≥28).

#### Disease-related characteristics

We chose methadone use, infection route, disclosure status, CD4 cell count, symptom counts, duration since HIV diagnosis, duration of treatment, viral load and the 30 most common symptoms for now according to the literature review and clinical experience. Years since HIV diagnosis, duration of treatment, CD4 cell count and viral load from database and clinic records. Disclosure status, infection route, and symptoms were obtained through patient self-reporting. If there were missing data in the database or clinic records, the investigator asked the patient for relevant information.

#### Health-related quality of life

HRQoL was assessed by the Medical Outcomes Study HIV Survey (MOS-HIV), which is a brief, comprehensive measure of health-related quality of life used extensively in HIV/AIDS (33). It consists of 35 items and measures 10 dimensions, including general health, physical function, role function, social function, cognitive function, pain, mental health, energy/fatigue, health distress, and quality of life. Of these 10 domains, 8 were multi-item and 2 (Social Function and Quality of Life) consisted of a single item. Ten subdimension scores and two summary dimensions (physical health summary score, PHS and mental health summary score, MHS) were obtained by adding the raw item scores of the respective scales and then transforming them into a 0–100 scale, with higher scores indicating better functioning and wellbeing. The simplified Chinese version of the MOS-HIV questionnaire has been reported to have good validity and reliability (34, 35). The Cronbach's α of the PHS and MHS scales was 0.87 and 0.89, respectively. The Cronbach's α for individual dimensions was >0.70 (34).

### Statistical analyses

Data were inputted by two researchers through EpiData 3.1, and all statistical analyses were performed using SPSS 24.0. A *P-*value of <0.05 was considered statistically significant. Descriptive statistics analysis was performed by mean ± standard deviation (SD) and median, interquartile range (IQR), frequency, and constituent ratio, as appropriate. We divided PHS and MHS scores into dichotomous variables by 50, in which scores above or below 50 can be considered better or worse HRQoL (36). Univariate analysis χ^2^-test for categorical data and Spearman's correlation for continuous data were performed to examine the quality of life of PLWH with different demographic characteristics and disease-related characteristics. Intragroup comparisons for multiple categorical variables were also performed by Fisher's exact test. Binary logistic regression analysis was conducted to explore factors associated with HRQoL in PLWH. Because there is no universal consensus in choosing predictor variables in a multivariable logistic regression analysis, we included all the variables with *P* ≤ 0.2 in the univariate analysis and all clinically significant variables regardless of their *P*-values, based on the literature evidence. The Hosmer–Lemeshow goodness-of-fit statistic with a *P*-value > 0.05 was considered a well-fitting regression model, and the percentage of the variability predicted by the model was explained by the Nagelkerke *R*^2^.

## Results

### Sociodemographic and disease-related characteristics of participants

Finally, 410 participants were enrolled in the study. However, A total of 9 questionnaires with missing values >20% were excluded, and the final number of valid questionnaires collected was 401. Among the participants, 64.7% (*n* = 258) were male and 35.2% (*n* = 141) were female. Their mean (*SD*) age was 50.02 (15.06) years. Most participants were of Han ethnicity (*n* = 340, 85.4%), married (*n* = 227, 58.2%), and employed (*n* = 304, 76.9%). Nearly half of the participants were living in rural areas (*n* = 209, 53.3%). The sociodemographic characteristics of the participants and the HRQoL in different groups are presented in Table 1.

**Table 1:** The socio-demographic of total samples, good or bad PHS/MHS groups of people living with HIV/AIDS (PLWH) (*N* = 401).

**Variables**	**Total (*N* = 401)**	**PHS**				**MHS**			

		**Bad (**<**50)**	**Good (**≥**50)**	*c*^2^**/ Spearman's** ρ	***p***	**Bad (**<**50)**	**Good (**≥**50)**	*c*^2^**/ Spearman's** ρ	***p***
Sex				1.87	0.171			0.09	0.760
male	258	59	199			93	165		
Female	141	41	100			53	88		
Age groups				9.896	**0.019**			1.32	0.720
18–45	168	31	137			60	108		
45–60	117	29	88			46	71		
≥60	115	40	75			39	76		
Age, mean (SD)^a^	50.02 (15.06)	48.35 (14.75)	55.03 (14.96)	−0.19	**<0.001**	50.21 (14.09)	49.69 (15.34)	0.02	0.741
Marital status				4.64	0.200			2.95	0.399
Unmarried	56	11	45			26	30		
Cohabitation or married	227	52	175			80	147		
Separation or divorce	67	23	44			23	44		
Bereaved spouse	40	11	29			13	27		
Ethnicity				1.45	0.485				
Han	340	85	255						
Yi	55	16	39						
Others	3	0	3						
Education level				12.22	**0.016**			18.81	**0.001**
Illiterate	42	17	25			14	28		
Primary school	146	42	104			57	89		
Senior high	130	30	100			38	92		
Junior high or technical secondary school	49	8	41			15	34		
Junior college or Bachelor's degree	30	3	27			21	9		
Sexual orientation				7.18	0.067			5.05	0.168
Homosexualit	22	7	15			8	14		
Heterosexuality y	339	84	255			119	220		
Bisexual	10	0	10			6	4		
Asexuality	10	5	5			6	4		
Religious beliefs				0.26	0.610			0.01	0.915
Yes	46	13	33			17	29		
No	343	85	258			124	219		
Occupation				5.86	0.053			1.5	0.471
Employed	304	67	237			107	197		
Unemployed	48	15	33			19	24		
Retirement	43	16	27			19	29		
Residence				2.1	0.147			1.22	0.270
Cities and towns	183	40	143			72	111		
Rural	209	59	150			71	138		
Per capita monthly income/yuan				3.46	0.630			10.64	0.059
0–499	88	25	63			26	62		
500–999	82	23	59			22	60		
1,000–1,499	64	16	48			27	37		
1,500–1,999	32	7	25			12	20		
2,000–2,499	39	11	28			18	21		
≥2,500	83	15	68			38	45		
Smoking habits				0.04	0.979			0.46	0.793
Never Smoking	249	62	187			94	155		
Smoking	124	32	92			43	81		
Quitted smoking	27	7	10			9	18		
Drinking habits				1.83	0.177			0.51	0.477
Yes	91	18	73			36	55		
NO	310	83	227			110	200		
BMI				20.81	**<0.001**			0.80	0.849
Underweight (<18.5)	49	19	30			16	33		
healthy weight (18.5–24.9)	235	68	167			86	149		
Overweight (25–27.9)	83	6	77			30	53		
Obese (≥28)	23	7	16			10	13		

^χ2^: Chi square value; Spearman's ρ: Spearman's correlation.

a Spearman's correlation was used to examine the good or bad PHS/MHS.

The bold values indicate that the *P* value is less than 0.05 and statistically significant.

### HIV-related variables

The mean duration after being diagnosed with HIV was 3.7 (SD = 3.3) years, ranging from 1 month to 30 years. All participants received ART with an average duration of 2.8 (SD = 2.4) years. Most of the participants' (*n* = 284, 72.6%) immune systems were weakened by HIV, while more than half of the patients had viral suppression (*n* = 249, 75.2%) and disclosed the HIV-infected condition (*n* = 287, 74.1%). The most common form of infection was sexual contact (*n* = 184, 47.1%). Most participants reported that they had more than one symptom, and only 14.5% (*n* = 58) of people indicated that they were not bothered by HIV-related symptoms (Table 2).

**Table 2:** HIV-related variables (*N* = 401).

**HIV-related variables**	**Total (*N* = 401)**	**PHS**				**MHS**			
		**Good (**≥**50)**	**Bad (**<**50)**	χ^2^**/ Spearman's** ρ	***p*** **-value**	**Good (**≥**50)**	**Bad (**<**50)**	χ^2^**/ Spearman's** ρ	***p*** **-value**
Methadone				10.83	**0.003**			0.06	1.000
Yes	10	7	3			4	6		
No	388	94	295			141	248		
Infection route				3.85	0.279			4.01	0.261
Sexual contact	184	42	142			59	125		
Mother-to-child transmission	1	10	15			0	1		
Blood transmission	25	0	1			11	14		
Unknown	180	47	133			73	107		
Disclosure				0.39	0.535			2.88	0.90
Yes	287	75	212			44	56		
No	100	23	77			99	188		
CD4 cell count(cells/mm^3^)				15.60	**<0.001**			0.46	0.794
<200	6,616.8	26	40			26	40		
200–500	21,855.7	56	162			77	141		
>500	10,727.3	14	93			37	70		
Viral load (copies/ml)				9.99	**0.013**			8.42	**0.029**
<50	249	188	61			160	89		
50–9,999	65	55	10			45	20		
10,000–99,999	13	6	7			4	9		
>100,000	4	2	2			4	0		
Symptoms counts^a^				−0.28	**<0.001**			−0.09	0.056
Mean ± SD	4.64 ± 5.94								
Min–max	0–30								
Duration since HIV diagnose (years)^a^				0.01		0.815		0.03	0.544
Mean ± SD	3.71 ± 3.26								
Min–max	0–23								
Duration received ART (years)^a^				0.02	**0.020**			−0.02	0.760
Mean ± SD	2.81 ± 2.43								
Min–max	0–12								
PHS								2.99	0.084
Bad						44	57		
Good						102	198		
MHS				2.99	0.084				
Bad		44	102						
Good		57	198						

χ^2^: Chi square value; Spearman's ρ: Spearman's correlation.

a Spearman's correlation was used to examine the good or bad PHS/MHS.

The bold values indicate that the *P* value is less than 0.05 and statistically significant.

### Symptoms

The majority of participants suffered fewer than 10 symptoms (Table 2). The top 5 symptoms reported by patients were insomnia (32.90%), fatigue (29.00%), forgetfulness (28.10%), joint pain (24.60%) and dry mouth (24.40%). Most participants' symptoms were light (Table 3). The least reported symptom was mouth ulcers. We found that anxiety and depression were not the most frequent symptoms among PLWH. In this study, the number of patients who reported having symptoms of anxiety and depression was 68 (16.6%) and 46 (11.3%), respectively.

**Table 3:** Symptom statistics table (*n* = 401).

**Symptom**	***N***	**%**
Insomnia	135	0.34
Fatigue	119	0.30
Forgetfulness	115	0.29
Joint pain	101	0.25
Dry-mouth	100	0.25
Muscle pain	91	0.23
Headache	88	0.22
Rash	83	0.21
Weakness	81	0.20
Thirst	77	0.19
Shortness of breath after activity	76	0.19
Inattentiveness	76	0.19
Anxiety	68	0.17
Decrease in appetite	65	0.16
Daytime sweating	59	0.15
Night sweating	52	0.13
dilute stool	48	0.12
Depression	46	0.11
Mouth ulcers	46	0.11
Nauseating	45	0.11
Diarrhea	44	0.11
Fever	40	0.10
Abdominal pain	39	0.10
Bloating	38	0.09
Fear	32	0.08
Vomit	32	0.08
Inability to adapt	31	0.08
Respite	28	0.07
Chills	26	0.06
Shortness of breath	25	0.06

### HRQoL of participants

The Cronbach's α coefficient for the MOS-HIV in this study was 0.659. According to the questionnaire, the HRQoL of the participants in terms of PHS, MHS and 10-dimensional scores are presented in Table 4. The mean physical health summary (PHS) and mental health summary (MHS) scores were 53.66 (6.81) and 51.31 (7.66), respectively, and there were 300 (74.8%) participants with good PHS and 255 (63.6%) participants with good MHS based on the standardized mean score of 50 (36). Of the 10 dimensions, the highest mean was found in the role functioning dimension (87.39 ± 29.37), while the lowest mean was found in the general health perceptions subscale (61.51 ± 21.37). There was no significant correlation between PHS and MHS (Spearman's correlation = 0.20; *P* = 0.017) and no significant correlation between PHS and MHS rank material, which was divided by 50 to be considered a good or poor health situation (Pearson chi-square = 2.99, *P* = 0.084).

**Table 4:** HRQoL in patients with HIV: summary scores and dimension scores (*n* = 401).

	**Median (IQR)**	**Mean (SD)**
**Summary scores**		
Physical health summary score	55.1 (49.9–58.7)	53.6 (6.8)
Mental health summary score	53.0 (46.9–57.0)	51.3 (7.6)
**Dimension scores**		
General health perception	60.0 (45.0–75.0)	61.5 (21.3)
Physical functioning	91.7 (83.3–100.0)	87.2 (16.9)
Role functioning	100.0 (100.0–100.0)	87.3 (29.3)
Social functioning	100.0 (60.0–100.0)	82.9 (23.8)
Cognitive functioning	80.0 (70.0–92.5)	79.7 (16.0)
Pain	88.8 (77.8–100.0)	84.1 (17.5)
Mental health	76.0 (60.0–88.0)	74.5 (16.9)
Energy/fatigue	65.0 (60.0–75.0)	64.8 (11.7)
Health distress	95.0 (75.0–100.0)	84.2 (20.4)
Quality of life	75.0 (50.0–75.0)	65.8 (16.1)

### Factors associated with HRQoL

In the univariate analysis (Tables 1, 2), the PHS was significantly higher in participants who were younger (*p* = 0.036), had better education (*p* = 0.005), had fewer symptoms of HIV (*p* < 0.001), had a CD4 level >500^cells/mm3^ (*p* = 0.004), had a viral load <10,000 copies/ml, did not use methadone (*p* = 0.001) and had a BMI between 18.5 and 27.9 (*p* < 0.001). Significantly higher MHS scores were reported by people with higher education levels (*p* = 0.001) and fewer symptoms. However, there was no correlation between disclosure, sex, sexual orientation and HRQoL score.

The association of the predictor variables with the PHS and MHS categories were explored using multivariate analysis and multivariable logistic regression analyses and are presented in Table 5.

**Table 5:** Factors associated with health-related quality of life in the multivariable logistic regression (*N* = 401).

**Outcomes**	**Variables**	**OR**	**95%CI**		***P*-value**
			**Upper limits**	**Lower limits**	
PHS	Symptoms counts	0.90	0.86	0.94	0.000
	BMI	0.001
	Underweight vs. healthy weight (reference)	1.09	0.37	3.25	0.871
	Overweight vs. healthy weight (reference)	0.55	0.16	1.88	0.34
	Obese vs. healthy weight (reference)	5.62	1.44	21.96	0.013
	CD4 counts	0.037
	200–500 vs. < 200 (reference)	0.01	0.33	0.14	0.769
	>500 vs. < 200 (reference)	0.08	0.53	0.26	1.088
	Methadone	0.07	0.01	0.45	0.005
	Age	0.97	0.95	0.99	0.012
	Education level	0.022
	Primary school vs. illiterate (reference)	0.09	0.02	0.49	0.005
	Senior high vs. illiterate (reference)	0.18	0.04	0.89	0.036
	Junior high or technical secondary school vs. literate (reference)	0.33	0.07	1.56	0.161
	Junior college or Bachelor's degree vs. illiterate (reference)	0.30	0.05	1.76	0.184
MHS	Symptoms counts	0.96	0.92	1.00	0.043
	Education level	0.002
	Primary school vs. illiterate (reference)	5.21	1.75	15.53	0.003
	Senior high vs. illiterate (reference)	3.64	1.48	8.98	0.005
	Junior high or technical secondary school vs. illiterate (reference)	6.64	2.62	16.85	0.000
	Junior college or Bachelor's degree vs. illiterate (reference)	5.53	1.90	16.11	0.002

Only educational level and symptom counts were both significant in PHS and MHS in the univariable analysis and multivariable logistic regression analyses. However, there was no significant correlation between PHS and MHS by Spearman's correlation or χ^2^-test. Youth population, higher education, health BMI level, CD4 level ≥500 cells/mm^3^, fewer symptoms, not using methadone and shorter time receiving ART were associated with good PHS in the univariable analysis, whereas only education level and symptom counts were associated with MHS. Some variables significantly associated with PHS and MHS in the univariate analyses were not significant in the multivariable models (Table 4). The Hosmer–Lemeshow goodness-offit test *p*-values were 0.628 and 1 for the PHS and MHS models constructed, respectively, suggestive of well-fitting models. It is assumed that 33.2% of the variability in the PHS (Nagelkerke *R*^2^ = 0.33) and 7% of the variability in the MHS (Nagelkerke *R*^2^ = 0.07) is explained by these models.

## Discussion

The results of the study showed that the total physical health score (PHS) and mental health score (MHS) of HIV/AIDS in Sichuan Province were 53.6 ± 6.8 and 51.3 ± 7.7, which were higher than those in previous studies surveyed in other areas of China (37–40). The reason may be that some time has passed since the previous studies were conducted. The impact of this illness on their quality of life is substantially lower than a few years ago because of the significant improvement in mortality and morbidity in PLWH with the implementation of ART (21). With the implementation of the “Four Free and One Care” policy, PLWH do not need to pay for medications, testing and counseling, can even obtain relief money from the government. Antiviral treatment and the four-free care policy help PLWH in poor areas of China receive better treatment, which helps to control disease and improve their quality of life.

The dimensions that scored higher were role function, physical function and health distress, which is similar to other studies (21, 41, 42). This is probably because HIV/AIDS can be controlled like any other chronic disease with the implementation of ART (41). The rate of severe illness decreased, and patients did not suffer significant impairment in physical or social functioning. The participants reported lower scores in the quality of life, energy/fatigue and general health perception dimensions, consistent with the results of previous studies (21, 41, 43). The lowest mean score was 61.52 for general health perception. The highest score in the energy/fatigue dimension is 95, and only in this dimension did no patient report a full score, 100. This result indicated that HIV-infected peoples' quality of life was still impaired and that each participant's energy was affected to varying degrees. Fatigue is the second most common symptom, with a 29% reporting rate. The causes of fatigue are complex. The disease itself, comorbidities, drug effects and even psychological factors may cause fatigue (41, 44, 45). We suggest that fatigue management and self-perception of health promotion are areas that need further attention for researchers.

The study revealed that education level was significantly associated with both the PHS and MHS of PLWH. The vast majority (80.1%) of participants in our study had only received a junior high school education or less. A further 10.5% were illiterate. Consistent with the findings of previous studies (42, 46, 47), educational level was a protective factor of HRQoL. Moreover, having no formal education is a barrier to accessing health services and increasing PLWH vulnerability (48). This result was also confirmed in this study, even if PLWH with primary education had a much lower risk of low quality of life than illiterate PLWH. The impact of education level on PLWH is multifaceted. It plays an important role in the spread and prevalence of HIV (49) and is more likely to lead to risky sexual behavior (50). Well-educated PLWH were more likely to show good knowledge, positive attitudes toward HIV/AIDS (48) and good social support (47) and cognitive level (42, 46), which leads to better self-management and health outcomes. Although education level is difficult to change, we recommend that more health information and education be provided through brochures, bulletin boards, and public online platforms. More tailored patient education should be conducted during the patient's hospitalization and at follow-up visits.

Symptoms of HIV have also been reported to be associated with HRQoL in many previous studies (22, 32, 51, 52), and more serious complications can lead to poor quality of life. In our study, we found that the number of symptoms was a risk factor for HRQoL, both in PHS and MHS. The most common symptoms reported by the participants in our study were sleep disturbances, fatigue, forgetfulness, joint pain and dry mouth. Sleep disturbances, fatigue, and forgetfulness have received extensive attention in previous studies (35, 53, 54). There are many factors that influence the symptoms, including the impact of AIDS on the immune system (53, 54), the adverse effects of medication (35, 55, 56) and psychosocial factors (45, 55, 57). Many studies have revealed that joint pain (58–62) and xerostomia are also common symptoms in PLWH (63, 64). In a cross-sectional survey of 195 PLWH conducted in Brazil, 40% of patients reported dry mouth symptoms (63). In a cross-sectional survey of 312 PLWH conducted in Italy, 34% reported joint pain (65). They have all been shown to have a negative impact on the QOL of PLWH (63, 65, 66). Possible causes are joint pain and salivary gland hypofunction due to ART (59, 64, 67). It has also been suggested that joint pain may be related to inflammatory responses mediated by inflammatory factors, but the exact mechanisms need to be further elucidated (68). This suggests that we need to include a full range of physical, psychological, and social care in future studies to reduce the impact of symptoms on the quality of life of PLWH and implement proven effective interventions in health services. Meanwhile, PLWH's joint pain and dry mouth need more attention by researchers and medical services team members.

Age was an independent risk factor that was significantly associated with the physical health of PLWH, which was similar to other studies (69, 70) but different from George's research (21). It may be that older PLWH experience a greater burden of age-related comorbidities, poorer social determinants of health and even ageism (71). Murzin's team (71) showed that ageism transcended multiple interactions and environments from dating to healthcare and community services to society because of social determinants, health provider issues and structural challenges. Therefore, we suggest that the government should pay more attention to older people, especially in the advent of an aging society.

This study showed a significant relationship between HRQoL PHS and CD4 lymphocyte counts. As CD4 lymphocyte counts increased, physical HRQoL improved. The findings were consistent with the results of other studies (31, 72, 73). A few studies have reported that lower CD4 counts may affect physical health (74–76). CD4+ cell count is an indicator of clinical progression and can reflect the impact of therapeutic efforts. Because of the “Four Frees and One Care” Policy, all HIV/AIDS patients in China can receive free ART in China. However, a relatively low CD4+ cell count of participants was also found in our study. This suggests that strategies to enhance medication adherence may be needed.

BMI is considered to be an important factor associated with patients' quality of life. In our study, both malnutrition and obesity may have contributed to the impairment of physical health. This is consistent with previous studies (77, 78), and some studies showed that obesity or malnutrition affects adult PLWH muscle strength (79) and the risk of frailty (80, 81) and comorbidities in PLWH followed up for 12 years (78). Even, BMI can be a predictor tool for death risk in Ethiopian adults living with HIV on ART (82). However, it is noteworthy that the PHS scores of the overweight group were the highest, even exceeding the healthy BMI level. Obesity may be due to a lack of physical activity and poor lifestyle (21), while underweight may be associated with disease consumption (77). We need more research to reveal whether the burden of being overweight will or will not affect patients' psychosocial functioning and how it could happen.

In addition, only 75.2% of participants in this study achieved viral suppression, which is much lower than the 92.4% in Beijing (78) but close to the figure for Liangshan, Sichuan (79). This may be attributed to the relatively short duration of HIV diagnosis and antiretroviral treatment in most of our participates. In addition, the comparison of data from two districts in China also illustrates the large differences in treatment effectiveness between regions. In particular, there is the largest base of HIV-infected patients in Sichuan Province. To achieve the “95 95 95” targets, there is a greater need for more targeted programs in less economically developed regions to explore critical influencing factors and efficient management interventions. Rationalize the allocation of limited resources to the whole process of HIV/AIDS management.

Although some studies have shown that women with HIV/AIDS have a higher quality of life than men (80, 81), we did not find any differences in quality of life between male and female HIV/AIDS patients. The difference might be due to the study population, sampling method, and measurement tools used to assess HRQoL among studies. A cross-sectional study of sex differences in quality of life among PLWH found that total physical health scores and mental health scores among PLWH were not statistically significant by sex (82).

Disclosure of HIV infection in this study was 71.6%, higher than other studies (21, 83). The majority of participants chose to disclose to their spouses (58.9%) and children (46.7%), and a small proportion disclosed the disease to parents (19.6%) and friends (7.4%). This may be one of the reasons why the MHS score in this study was higher than that in other studies.

One thing that should not be overlooked in our study is that PLWH explain only 7% of the PHS, and only two factors are included in the final multivariate regression. The reason may be that the sample was a general adult PLWH population with higher psychological scores than those reported in other literature (84, 85), and fewer people reported anxiety (17%) and depression (11%) among the symptom questionnaires, which indicates a relatively high level of mental health in this study sample. Second, nearly half of the PLWH in this sample had received only primary education and below, and their cognitive level of mental health might not be sufficient (86). Last, previous studies have shown that disclosure, sexual orientation and other factors have a significant impact on the mental health of PLWH (87), the level of disclosure in this sample population is higher than that in previous studies (21, 83), and the sexual orientation is mostly heterosexual. It would be easier to tap into special populations such as men who have sex with men (MSM), sexual service providers or adolescent groups to identify the relevant influencing factors.

There were limitations in our study that should be acknowledged. First, it is a population with a relatively recent history of antiretroviral treatment but with many symptoms that seem more related to treatments used in the past. However, the type of treatment and regimens used are not available. We were unable to analyze whether the patient-reported symptom presentation was related. Second, the study was conducted in a single city in Sichuan. Future studies need to be conducted in other Sichuan provinces and even nationwide to verify whether there are differences in PLWH quality of life across geographic areas. Third, we could not determine the exact causality between HRQoL and the related factors due to the cross-sectional design. Therefore, there is a need for longitudinal research and multicenter studies to confirm our findings and explore their causality.

## Conclusion

In conclusion, the HRQoL of PLWH in Panzhihua improved compared to previous studies. Alleviating symptoms of HIV and preventing comorbidities, especially insomnia and fatigue, are important for patient life treatment. We recommend that further research should be conducted to explore factors affecting quality of life so that we can develop more comprehensive interventions to prevent HIV/AIDS. Moreover, more care and support should be given to patients, especially for those with lower educational attainment, unhealthy body mass index, more symptomatic presentation and older age.

## Data availability statement

The original contributions presented in the study are included in the article/supplementary material, further inquiries can be directed to the corresponding author.

## Ethics statement

The studies involving human participants were reviewed and approved by Sichuan University Medical Ethics Committee. The patients/participants provided their written informed consent to participate in this study. Written informed consent was obtained from the individual(s) for the publication of any potentially identifiable images or data included in this article.

## Author contributions

HZ: writing manuscripts and data processing. FW: data entry and writing manuscript. YS, ZN, and HC: draft touch-ups and guidance on writing. All authors contributed to the article and approved the submitted version.
